# Cytomegalovirus Viral Load Kinetics in Patients with HIV/AIDS Admitted to a Medical Intensive Care Unit: A Case for Pre-Emptive Therapy

**DOI:** 10.1371/journal.pone.0093702

**Published:** 2014-04-03

**Authors:** Simnikiwe H. Mayaphi, Marieke Brauer, Daniel M. Morobadi, Ahmad H. Mazanderani, Rendani T. Mafuyeka, Steve A. S. Olorunju, Gregory R. Tintinger, Anton Stoltz

**Affiliations:** 1 Department of Medical Virology, University of Pretoria/National Health Laboratory Service - Tshwane Academic Division (NHLS-TAD), Pretoria, South Africa; 2 Biostatistics unit, Medical Research Council, Pretoria, South Africa; 3 Department of Internal Medicine, University of Pretoria, Pretoria, South Africa; University of Regensburg, Germany

## Abstract

**Background:**

Cytomegalovirus (CMV) infection is associated with severe diseases in immunosuppressed patients; however, there is a lack of data for pre-emptive therapy in patients with HIV/AIDS.

**Method:**

This was a retrospective study, which enrolled patients diagnosed with HIV/AIDS (CD4<200 cells/μl), who had detectable CMV viral load (VL) during their stay in an adult medical intensive care unit between 2009–2012.

**Results:**

After screening 82 patients’ records, 41 patients met the enrolment criteria. Their median age was 37 (interquartile range [IQR]: 31–46), and median CD4 count was 29 cells/μl (IQR: 5–55). Sixteen patients (39%) had serial measurements of CMV VL before treatment with ganciclovir. Patients whose baseline CMV VL values were between 1,000–3,000 copies/ml had significantly higher values (median of 14,650 copies/ml) on follow-up testing done 4–12 days later. Those with undetectable VLs at baseline testing had detectable VLs (median of 1,590 copies/ml) mostly within 20 days of follow-up testing. Patients who had VLs >1,000 copies/ml at baseline testing had significantly higher mortality compared to those who had <1,000 copies/ml {hazard ratio of 3.46, p = 0.003 [95% confidence interval (CI): 1.55–7.71]}. Analysis of the highest CMV VL per patient showed that patients who had VLs of >5,100 copies/ml and did not receive ganciclovir had 100% mortality compared to 58% mortality in those who received ganciclovir at VLs of >5,100 copies/ml, 50% mortality in those who were not treated and had low VLs of <5,100 copies/ml, and 44% mortality in those who had ganciclovir treatment at VLs of <5,100 copies/ml (p = 0.084, 0.046, 0.037, respectively).

**Conclusion:**

This study showed a significantly increased mortality in patients with HIV/AIDS who had high CMV VLs, and suggests that a threshold value of 1,000 copies/ml may be appropriate for pre-emptive treatment in this group.

## Introduction

The prevalence of human cytomegalovirus (CMV) in adults is approximately 60% and 95% in developed and developing countries, respectively [1]. CMV is responsible for a variety of clinical diseases in immunosuppressed patients, which include retinitis, pneumonitis, encephalitis, oesophagitis, colitis, hepatitis and others [2]. It is a betaherpesvirus that establishes latency in mature monocyte-derived macrophages and dendritic cells as well as in CD34+ bone marrow progenitor cells, with reactivation only occurring in mature cells [3].

Ganciclovir is the drug of choice for the treatment of CMV, but valganciclovir can be used where oral administration is possible [4]. Common side effects of ganciclovir such as neutropaenia and anaemia can complicate patient management. For this reason, it is very important to properly diagnose or predict CMV disease in order to avoid unnecessary treatment and side effects from these drugs. The principle of pre-emptive therapy is based on identifying and treating those at high risk of disease in order to avoid CMV-related morbidity and mortality [3].

Diagnosis of CMV disease is complicated by the fact that this virus can reactivate and be shed in various body fluids without causing disease [5]. It is, therefore, always important to differentiate CMV infection from disease or identify those at high risk of disease. Laboratory assays that are commonly used for the diagnosis or prediction of CMV disease are CMV PCR (qualitative/quantitative), pp65 antigen, and histology/cytology (usually from biopsy samples). The latter is regarded as the gold standard for CMV disease as it shows organ invasion by CMV. However, it is not always possible to obtain biopsy samples as this procedure is more invasive [5]. CMV viral load (CMV VL) assays from blood samples are commonly used for prediction of CMV disease as they have high sensitivity, and are simpler to perform and interpret than the other tests [5], [6]. Published data from transplant patients show that a CMV VL of 10000 copies/ml or above is associated with high risk of CMV disease in solid organ transplant patients, while a 1000 copies/ml threshold is used for stem cell transplant patients as they are severely immunosuppressed. These values are used for pre-emptive therapy in these groups of patients [4], [7]. Some experts have shown a threshold of 2600 copies/ml as appropriate for pre-emptive therapy even in solid organ transplant patients at low risk of CMV reactivation [8].

There is a paucity of data for treatment of CMV infection in patients with HIV/AIDS even though these patients have a similar spectrum of CMV disease manifestations as transplant patients. As a result, extrapolation of transplant data is often used for management of CMV infection in patients with HIV/AIDS. However, it is not known whether patients with HIV/AIDS are as immunosuppressed as solid organ or stem cell transplant patients, thus making the selection of an appropriate CMV VL threshold difficult for this group of patients. Trends observed (unpublished data) in our adult medical intensive care unit (ICU) have shown that the use of a CMV VL threshold of ≥10000 copies/ml for CMV treatment is associated with a high mortality. Consequently, a lower threshold of 1000 copies/ml has been adopted for use in our ICU from around mid-year 2011. The aim of this study was to evaluate the CMV VL kinetics in patients with HIV/AIDS in an ICU environment.

## Materials and Methods

This was a retrospective study, which reviewed medical records of patients with CMV VL test results who were admitted in adult medical ICU at Steve Biko Academic hospital between January 2009 to December 2012. The University of Pretoria’s Faculty of Health Research Ethics committee granted ethics approval for the study protocol, including approval for authors to access patients’ records as almost all patients were no longer hospitalised at the time this study was conducted. Approval for accessing patients’ records was also obtained from the superintendent of Steve Biko Academic hospital. Data from the records was anonymized and de-identified prior to analysis in order to ensure patient confidentiality. The study participants were identified through a search in the National Health Laboratory Service (NHLS) database for patients who had a CMV VL test while in ICU. Standard data capturing forms were used to collect patient demographics and clinical data from the records. These forms assessed the following information: dates of admission, discharge or death in ICU; patient diagnosis; ganciclovir use; other drug use (e.g. antibiotics, antivirals, steroids); and timing of antiretroviral initiation (before or after admission). The target group for enrolment were patients with AIDS as defined by CD4 count <200 cells/μl.

Laboratory information was obtained from the NHLS database, and this included CMV VL and serology, HIV tests, CD4 count, *Pneumocystis jirovecii* immunofluorescence, tuberculosis (TB), microbiology culture, and post-mortem results. Confirmed TB infections were based on positive TB culture and/or PCR results. Dates of CMV VL results and the number of these results per patient were documented. CMV VL testing was done on plasma samples using the following assays: Affigene assay (Cepheid, Solna, Sweden) with a limit of detection (LOD) of 235 copies/ml, and R-gene assay (Argene SA, Varilhes, France) with an LOD of 50 copies/ml, and both assays were run on the LightCycler instrument (Roche, Mannheim, Germany). The CMV VL testing was done in three different laboratories, all of which participated in international quality assurance schemes for CMV VL, and two were also approved by the local laboratory accrediting body (South African National Accreditation System). All the study information was collected by qualified medical doctors.

Intravenous ganciclovir was used at a dose of 5 mg/kg twice a day for 21 days, with dose adjustment for patients with renal dysfunction. All the CMV VL analysis in this study was done before treatment initiation with ganciclovir (i.e. for those who received treatment).

### Statistical Analysis

A descriptive analysis was used to present summary statistics (mean, standard deviation, standard error, median and 95% confidence intervals) for the parameters. This was followed by a comparison between the groups using two sample independent t-tests for proportions. Non-parametric survival analysis presenting Kaplan-Meier curve was performed to assess hazard ratios and association of different CMV VL values with mortality. Univariate logistic regression analysis was performed to assess the association of individual variable (age, CD4 count, CMV VL, confirmed TB infections and steroids) with mortality. In addition, multivariable logistic regression was undertaken, adjusting for age, CD4, and ganciclovir use. All the statistics was performed on the STATA version 12.1 software (StataCorp LP, College Station, TX, USA), and survival analysis was also performed on MedCalc version 12.7.0 software (Acacialaan, Ostend, Belgium). A p-value of <0.05 was considered statistically significant.

## Results

**Figure 1:**
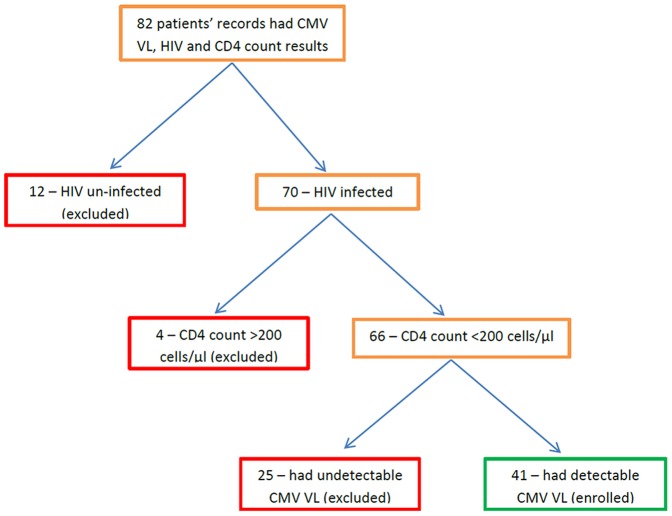
Algorithm showing the selection of study participants. Study participants were selected after a review of records and laboratory results for all the patients who had CMV VL results during ICU stay, within the study period. CMV VL = cytomegalovirus viral load.

During the study period, 82 patients were tested for CMV VL while in ICU, and also had HIV and CD4 results available. Of these patients, only 41 met the enrolment criteria for this study, i.e. had a detectable CMV VL and a diagnosis of AIDS as defined by CD4 count <200 cells/μl (Fig. 1). The enrolled participants represented 62% (41/66) of patients with AIDS (Fig. 1). The 25 patients with HIV/AIDS who were excluded did not have detectable CMV VL during their stay in ICU.

The enrolled patients were predominantly black (90%), the remaining 10% were white; and 54% were males. The median age of all patients was 37 (IQR:31–46), and the median CD4 count was 29 cells/μl (IQR:5–55). The majority of patients (65.9%) were diagnosed with pulmonary diseases (mainly community acquired pneumonia) at the time of admission to ICU, followed by renal (14.6%) and central nervous system diseases (12.2%), and septicaemia (7.3%). None of these patients were diagnosed with CMV retinitis. Appropriate antibiotics were used for empiric therapy and later modified based on microbiology cultures. Most patients (78%, n = 32) were not on antiretroviral therapy (ART) at the time of admission to ICU, and 44% (n = 14) of these patients later received ART. The median stay in ICU from admission to discharge or death was 19 days (IQR:12–44).

**Figure 2:**
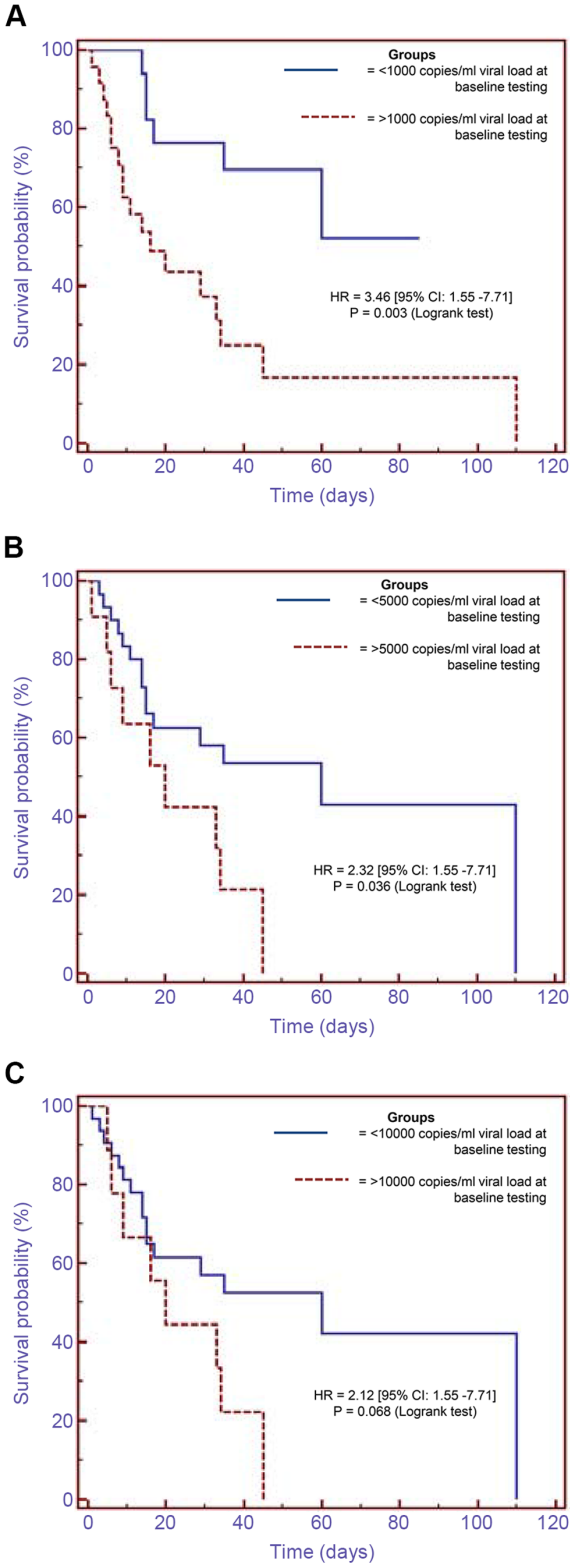
Kaplan-Meier curve analysis showing survival probalities between the groups of patients: (A) who had CMV viral loads <1000 copies/ml compared to those who had viral loads >1000 copies/ml at baseline testing, (B) who had CMV viral loads <5000 copies/ml compared to those who had viral loads >5000 copies/ml at baseline testing, (C) who had CMV viral loads <10000 copies/ml compared to those who had viral loads >10000 copies/ml at baseline testing. HR = hazard ratio, CI = confidence interval.

A large proportion of patients (71%, n = 29) had detectable viral loads at baseline testing with a median CMV VL of 3430 copies/ml (IQR:1380–16450), while others had undetectable viral loads. The latter eventually had detectable viral loads on subsequent testing. Sixteen patients (39%) had serial measurements of CMV VL before treatment with ganciclovir. Patients whose baseline CMV VL values were between 1000–3000 copies/ml had significantly higher values (median of 14650 copies/ml) on follow up testing done 4–12 days later (Table 1). Those with undetectable VLs at baseline testing had detectable VLs (median of 1590 copies/ml) mostly within 20 days of follow up testing (Table 2). Patients (n = 24) who had CMV VLs >1000 copies/ml at baseline testing had significantly higher mortality compared to those (n = 17) who had CMV VLs <1000 copies/ml (hazard ratio of 3.46, p = 0.003 [95% CI: 1.55–7.71]) (Fig. 2). The trend with higher mortality was also noticed when patients were stratified by a CMV VL of 5000 copies/ml at baseline testing, and no statistical significance noted with a CMV VL of 10000 copies/ml threshold (Fig. 2).

**Table 1:** Clinical characteristics of patients who had low CMV VL at baseline.

Study ID	Age	Sex	CD4 count	CMV VL results and dates (dd/mm/yy)		Ganciclovir initiation date (dd/mm/yy)	Death in ICU and dates (dd/mm/yy)	Group
1707	34	M	35	2430	19100	29/08/10	Yes	DT
				15/08/10	26/08/10		20/09/10	
689	49	F	48	1150	10200	29/07/10	No	DT
				20/07/10	24/07/10			
1851	23	F	149	2640	113000	N/A	Yes	nt-HVL
				07/08/10	16/08/10		17/08/10	
8540	67	M	57	1600	5690	N/A	Yes	nt-HVL
				20/01/11	01/02/11		05/02/11	

ID = identity, CMV VL = cytomegalovirus viral load, ICU = intensive care unit, DT = delayed treatment,

ntHVL = non-treatment and high viral load. N/A – ganciclovir was not administered as patients were admitted at a time when a 10000 copies/ml threshold for CMV treatment was used. For consistency, sample collection dates were used for CMV VL results.

**Table 2:** Clinical characteristics of patients who had undetectable CMV VL at baseline.

Study ID	Age	Sex	CD4 count	CMVVL results and dates(dd/mm/yy)			Ganciclovir initiation date (dd/mm/yy)	Death in ICU and dates (dd/mm/yy)	Group
76	30	M	6	LDL	2120	28200	17/02/11	No	DT
				31/01/11	01/02/11	09/02/11			
8063	40	M	9	LDL	3820	4860	17/06/11	No	ET
				21/05/11	10/06/11	17/06/11			
5558	14	F	5	LDL	1360	ND	24/11/11	Yes	ET
				27/10/10	23/11/10			20/12/10	
3938	30	F	8	LDL	LDL	3000	N/A	No	nt-LVL
				02/06/10	05/06/10	28/07/10			
7473	49	M	25	LDL	1590	ND	29/09/11	No	ET
				08/09/11	21/09/11				
6279	45	M	49	LDL	4770	ND	N/A	No	nt-LVL
				17/02/10	02/03/10				
3825	42	M	45	LDL	14600	ND	15/02/10	No	DT
				24/12/09	09/02/10				
2120	40	M	54	LDL	880	ND	N/A	Yes	nt-LVL
				17/03/10	26/03/10			28/03/10	
3032	47	F	75	LDL	LDL	4680	N/A	No	nt-LVL
				20/10/11	30/10/11	23/11/11			
276	49	F	29	LDL	835	ND	N/A	Yes	nt-LVL
				28/06/12	04/07/12			06/07/12	
6057	46	F	105	LDL	44	LDL	N/A	No	nt-LVL
				19/01/12	04/02/12	04/03/12			
3791	43	F	2	LDL	29200	ND	08/02/10	No	DT
				23/12/09	30/01/10				

ID = identity, CMV VL = cytomegalovirus viral load, ICU = intensive care unit, ET = early treatment,

DT = delayed treatment, nt-LVL = non-treatment and low viral load, ND = not done, N/A – ganciclovir was not administered as some of these patients were admitted at a time when a 10000 copies/ml threshold for CMV treatment was used, LDL = lower than detectable limit. For consistency, sample collection dates were used for CMV VL results.

When the highest CMV VLs that patients had during their ICU stay were analysed, it was noticed that patients who were not treated but had high VLs (nt-HVL) of >5100 copies/ml had 100% mortality compared to 58% mortality in those who received ganciclovir at >5100 copies/ml (delayed treatment [DT]), 50% mortality in those who were not treated and had low VLs (nt-LVL) of <5100 copies/ml, and 44% mortality in those who had ganciclovir treatment at <5100 copies/ml (early treatment [ET]) (p = 0.084, 0.046, 0.037, respectively) (Table 3). The reason for non-treatment is that some patients had undetectable or low CMV VL results at baseline testing at the time that we used 10000 copies/ml as a threshold for treatment, and only found to have significantly high CMV VL values on follow-up testing, which was at the time of death for some. Another reason is that some had very high CMV VL results at baseline testing and demised just after testing (more applicable to the nt-HVL group). The majority of patients (53.3%, n = 8) in the nt-LVL group had CMV VLs <1000 copies/ml. Steroid use was not statistically different amongst all these groups, even though it was slightly lower in the ET group (as compared with DT, nt-LVL and nt-HVL: p = 0.395, 0.476, 0.605, respectively). Confirmed TB infections were higher in the ET group (as compared with DT, nt-LVL and nt-HVL: p = 0.054, 0.627, 0.042, respectively), which had the lowest mortality (Table 3). *Pneumocystis jirovecii* immunofluorescence test was positive in 5 patients; however, most patients were treated or put on prophylaxis for pneumocystis pneumonia despite negative results as there is currently no test that completely excludes this disease [9]. Univariate logistic regression analysis showed that CMV VL was the only variable associated with mortality in this cohort, showing that patients with CMV VL >1000 copies/ml at baseline had 5 times greater odds (crude odds ratio of 5.5, p = 0.014 [95% CI: 1.4–21.4]) of dying compared to those with CMV VL <1000 copies/ml. Multivariable logistic regression still identified CMV VL as the only factor associated with mortality. The adjusted odds ratios were 5.44 [95% CI: 1.4–21.3], 5.81 [95% CI: 1.5–23.1], and 4.66 [95% CI: 1.1–19.6] after adjusting for age, CD4 count, and ganciclovir use, respectively. The odds ratio was 4.86 [95% CI: 1.1–21.6] after adjusting for all the variables together, and CMV VL association with mortality still remained significant (p = 0.038). Grade 4 neutropaenia was detected in 24% of patients who received ganciclovir.

**Table 3:** Characteristics of patients in different groups stratified by CMV VL of 5100 copies/ml and ganciclovir use.

	TREATMENT GROUPS		NON-TREATMENT GROUPS	
	ET	DT	nt-LVL	nt-HVL
	(<5100 copies/ml)	(>5100 copies/ml)	(<5100 copies/ml)	(>5100 copies/ml)
	n = 9	n = 12	n = 15	n = 5
Median age (IQR)	38	34	37	35
	(33–46)	(30–43)	(32–46)	(23–57)
Median CD4 count (IQR)	6	5.5	49	33
	(3–29)	(2–40)	(29–75)	(16–103)
Median CMV VL (IQR)†	2690	22150	990	100000
	(1590–4700)	(13000–35900)	(651–3000)	(6655–113000)
Confirmed TB infections	67%	25%	57%	25%
Steroid use	67%	83%	80%	80%
Mortality in ICU	44%	58%	50%	100%

ET = early treatment, DT = delayed treatment, nt-LVL = non-treatment and low viral load, nt-HVL = non-treatment and high viral load, TB = tuberculosis, ICU = intensive care unit.

† = highest CMV VL values per patient were used for this analysis.

Only two patients had post-mortem examinations performed, both of whom had CMV inclusion bodies on lung biopsy microscopy. Interestingly, one of these patients belonged to the nt-LVL group with a CMV VL of 880 copies/ml and was therefore not treated (Table 2 - patient 2120). The other patient belonged to the DT group and had a significantly high CMV VL of 21500 copies/ml and did receive ganciclovir treatment (data not shown). The low rate of post-mortem analysis is probably due to general decline in the use of this procedure in modern medicine [10], and failure to obtain consent from the relatives of the deceased.

CMV serology results were available for 7 patients (17%); all had the same profile of negative IgM and positive IgG, 4 of whom had high CMV VLs (median = 32700 copies/ml).

## Discussion

This study provides an analysis of the CMV VL kinetics in critically ill patients with HIV/AIDS. It is the first study to report on the CMV VL threshold value that may be appropriate for pre-emptive therapy in patients with AIDS.

The enrolled participants represented 62% (41/66) of the patients with AIDS, showing that CMV reactivation is very common in these patients in the ICU setting. The negative IgM and positive IgG CMV serology profile in all patients who had serology testing highlights the high prevalence of CMV in an African setting [11], indicating a high likelihood of CMV reactivation in patients with HIV/AIDS or other immunosuppressive conditions. Advanced immunosuppression could account for the negative CMV IgM results despite detectable CMV VLs in some patients [5], [12].

The CMV VLs were significantly higher in the group that had 100% mortality (nt-HVL group) compared to other groups (Table 3). This is consistent with data from other immunosuppressed groups of patients, which show that high CMV VLs are associated with CMV disease and high mortality [8], [13], [14]. In the treated groups, mortality was higher in those where therapy was delayed (DT group, with a median CMV VL of 22150 copies/ml) compared to the early therapy group (ET group, with a median CMV VL of 2690 copies/ml) (Table 3). Even though not statistically significant (p = 0.525), the clinical relevance of this finding cannot be ignored as some published data have shown that treatment started at higher CMV VL (>10000 copies/ml) is associated with delayed response or failure to control viraemia within 4 weeks [1], [15].

The significant mortality differences between the nt-HVL group as compared with nt-LVL and ET groups (p-values = 0.046, 0.037, respectively) suggest that pre-emptive therapy for CMV infection in patients with AIDS should be started at low CMV VLs in order to reduce morbidity and mortality. The higher mortality associated with a CMV VL >1000 copies/ml (Fig. 2), and the faster increase of low CMV VL values between 1000–3000 copies/ml to significantly higher values on follow up testing (Table 1) suggest that a CMV VL threshold of 1000 copies/ml may be appropriate to initiate pre-emptive CMV treatment in patients with HIV/AIDS in ICU. This is similar to the threshold VL that is commonly used in stem cell transplant patients at high risk of CMV reactivation [7]. This threshold is supported by the findings of recent studies, which noticed an association between CMV VL and high mortality in ambulatory patients with HIV/AIDS [16]–[18]. Death hazard ratios of 3.65 and 3.9 were reported by Fielding et al. and Boffi El Amari et al., respectively, when the CMV VL was ≥1000 copies/ml [16], [17]; while Durier et al. reported a death hazard ratio of 7.28 when the CMV VL was >500 copies/ml [18]. A recently published study, which employed a CMV VL threshold of 5000 copies/ml (and 3000 copies/ml for certain patients) showed that pre-emptive therapy prevents CMV end-organ disease by almost 25% in HIV infected patients with advanced immunosuppression [19]. This is; however, lower than 49% CMV disease risk reduction noted by Spector et al. during prophylactic use of oral ganciclovir in patients with AIDS [20]. The difference between the benefits of pre-emptive and prophylactic treatments between these two studies could be explained by a relatively higher CMV VL pre-emptive threshold of 5000 copies/ml, and by undetectable plasma CMV VL in some patients with CMV end-organ disease [21] who would have been missed in the pre-emptive treatment study. Some experts have shown that CMV VL values of <5000 copies/ml are associated with CMV disease. For instance, Rasmussen et al. reported that a CMV VL ≥32 copies/25 μl (i.e. ≥1280 copies/ml) in plasma, particularly when sustained, distinguished patients who developed CMV retinitis from those who did not [22]. The frequency of grade 4 neutropaenia (24%) noticed in this cohort is comparable to a frequency reported by Mizushima et al. for grade 3–4 leukocytopaenia in CMV/HIV co-infected patients receiving ganciclovir [19].

Some published data have shown evidence of CMV disease in autopsy or biopsy samples without detectable viraemia [21], [23], [24]. Therefore, an undetectable CMV VL in plasma does not exclude the presence of CMV disease. In this study patients who initially had undetectable CMV VL later had detectable viral loads in plasma during follow up testing (Table 2). A post-mortem report that showed CMV inclusion bodies on lung microscopy in a patient who had CMV VL results of 880 copies/ml indicates that by the time viraemia is detected in plasma there could already be established end-organ disease in some patients. This highlights the clinical significance of low CMV VL values below 1000 copies/ml in some patients. Where there is uncertainty regarding the relevance of low CMV VL values, follow up testing should be done, particularly if a patient’s clinical condition is not improving on appropriate therapy. The detection of CMV VLs during follow up in patients who initially had undetectable viral loads supports the practice of measuring CMV VL in the site of pathology where CMV infection may cause disease before it is detectable in the blood [25]–[27]. The site to be sampled should be determined by the patient’s clinical condition.

The majority of patients presented to ICU with pulmonary disease, mainly community acquired pneumonia. Although CMV can cause pneumonitis in immunosuppressed individuals [2], it is not known if it was the primary cause of pneumonia in this study as CMV disease was not proven by cytology/histology except in two patients who had post-mortem results. Interestingly, confirmed TB infections were higher in the ET group, which had the lowest mortality than the other groups; and the logistic regression analysis identified CMV VL as the only variable associated with mortality. This highlights that CMV infections play a more independent role towards mortality in the setting of high viral loads [13], [28], [29]. The absence of the CMV retinitis diagnosis in this cohort is surprising; however, this could have been missed as patients’ ophthalmology assessment was done without pupil dilatation, and not done by ophthalmologists. Also a recently published South African study showed a low incidence of CMV retinitis in the era of ART [30].

The strengths of this study include the availability of serial CMV VL measurements before treatment initiation in some patients and availability of clinical data (including time to discharge or death). Limitations include the small sample size, inclusion of patients with HIV/AIDS in an ICU setting only, unavailability of CMV VL testing in other samples other than plasma, and that post-mortem data was only available for two patients. The data on the rate of CMV VL increase from lower to higher values should be interpreted with caution as follow up testing was not done at the same regular intervals. Different CMV VL assays used in different laboratories could have influenced results as there was no standardisation of testing. Other opportunistic infectious diseases such as toxoplasmosis were not excluded.

## Conclusion

This study has for the first time provided data on CMV VL kinetics in patients with HIV/AIDS admitted to ICU. It showed a significantly increased mortality in patients with HIV/AIDS who had high CMV VLs, and suggests that a threshold value of 1000 copies/ml may be appropriate for pre-emptive treatment in this group of patients. Further studies are needed to confirm the relevance of this threshold.
